# Fatal Recurrent Splenic Artery Pseudoaneurysm Rupture Despite Prior Successful Embolization in Alcohol-Associated Chronic Pancreatitis: A Case Report

**DOI:** 10.3390/reports8040269

**Published:** 2025-12-18

**Authors:** Nawras Ibrahim, Stéphanie Ammari, Faiza Malik

**Affiliations:** HCA Houston Healthcare Clear Lake, University of Houston College of Medicine, Houston, TX 77598, USA

**Keywords:** alcohol use disorder, chronic pancreatitis, embolization failure, endovascular therapy, sentinel bleed, splenic artery pseudoaneurysm, recurrent splenic artery pseudoaneurysm

## Abstract

**Background and Clinical Significance:** Splenic artery pseudoaneurysm (SAP) is a rare but life-threatening complication of chronic pancreatitis. Although endovascular embolization achieves high technical success, recurrence and delayed rupture may occur, particularly in patients with ongoing pancreatic inflammation or alcohol use disorder (AUD). **Case Presentation:** A 47-year-old woman with alcohol-associated chronic pancreatitis presented with hematochezia, melena, and syncope. CT angiography revealed a 3.6 cm SAP adjacent to a 4.2 cm pancreatic head pseudocyst, and she underwent successful coil embolization. Despite initial stability, she relapsed into heavy alcohol use, experienced recurrent pancreatitis flares, and developed progressive multisystem comorbidities. Surveillance imaging up to three months post-embolization showed pseudocyst fluctuations without early recanalization, but long-term follow-up lapsed. Eight months after embolization, she presented in hemorrhagic shock from recurrent SAP rupture and died despite massive transfusion and emergent splenic artery ligation. **Conclusions:** Fatal SAP rupture may occur months after technically successful embolization. Sentinel bleeding, AUD relapse, and progressive systemic decline are critical warning signs. Structured post-embolization imaging and multidisciplinary management are essential to improve long-term outcomes.

## 1. Introduction

Splenic artery pseudoaneurysm (SAP) is a rare vascular complication of pancreatitis, seen in less than 1% of cases, but rupture carries a mortality rate of up to 80–90% if untreated [1,2]. SAP formation results from enzymatic erosion of the splenic arterial wall, often in association with pancreatic pseudocysts or chronic inflammation [3].

Endovascular embolization has become the first-line therapy, with immediate technical success reported in more than 95% of cases [4]. However, long-term failure, including recanalization, collateralization, or new pseudoaneurysm formation, can occur. This risk is particularly pronounced in the context of persistent pancreatic inflammation or ongoing etiological risk factors, such as alcohol use disorder (AUD) [5]. Despite high initial success rates, the long-term management strategy for these high-risk patients remains a clinical challenge, as evidenced by limited data on recurrence rates and optimal surveillance protocols [6,7,8,9].

We present a case of fatal recurrent SAP rupture eight months after initial coil embolization in a patient with alcohol-associated chronic pancreatitis. This case highlights the prognostic significance of a sentinel bleed, the critical impact of alcohol relapse, and the role of progressive systemic comorbidities in patient outcomes [6,7,8].

## 2. Case Presentation

**Figure 1:**
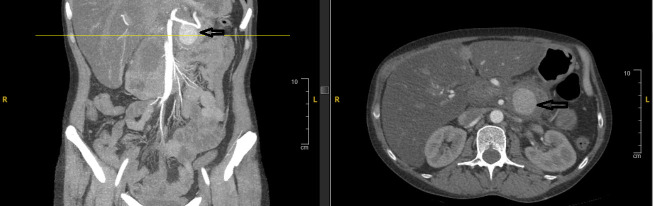
Contrast-enhanced CT angiography of the abdomen showed a 3.5 cm pseudoaneurysm developing from the splenic artery at the junction of the pancreatic body and tail (arrows) next to a 5.4 cm pancreatic head pseudocyst (no arrows).

**Figure 2:**
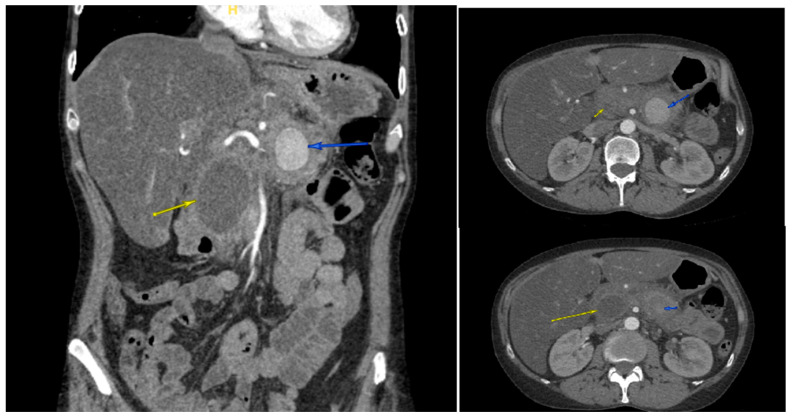
Contrast-enhanced CT angiography of the abdomen showed a 3.6 × 3.7 × 3.5 cm pseudoaneurysm (blue arrows), next to a 4.2 cm pancreatic head pseudocyst (yellow arrows).

A 47-year-old female with a remarkable history of alcohol-induced chronic pancreatitis and alcohol use disorder presented in June 2024 with symptoms including hematochezia, melena, and syncope. Upon her admission, she showed signs of low blood pressure and tachycardia. The results of laboratory tests showed a hemoglobin level of 7.1 g/dL and a serum lipase of 449 U/L. Given the patient’s hemodynamic instability and history of chronic pancreatitis, CT angiography (CTA) was performed urgently instead of initial esophagogastroduodenoscopy (EGD) to rule out vascular complications, revealing a 3.6 × 3.7 × 3.5 cm pseudoaneurysm developing from the splenic artery at the junction of the pancreatic body and tail (Figure 1), next to a 4.2 cm pancreatic head pseudocyst (Figure 2). The bleed was presumed to involve fistulization into the GI tract, potentially via the pancreatic duct (virsungorrhagia), though not directly visualized on initial or follow-up imaging.

**Figure 3:**
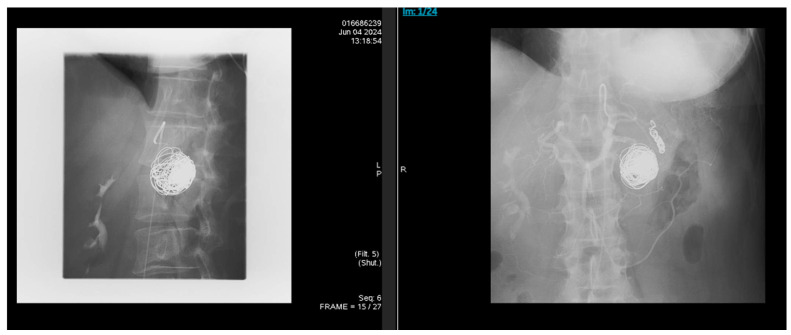
Following the procedure, angiography was done, and it showed total occlusion of the pseudoaneurysm sac and distal splenic artery segment, with preserved splenic perfusion via collaterals.

The patient had immediate visceral angiography with successful coil embolization of the splenic artery pseudoaneurysm. Covered stent placement was considered but not pursued due to anatomical constraints, including vessel tortuosity and caliber. Following the procedure, angiography confirmed total occlusion (Figure 3). Coil embolization targeted the pseudoaneurysm sac and involved proximal and distal occlusion of the affected splenic artery segment to prevent recanalization and backflow, resulting in loss of antegrade flow distal to the coils with preserved splenic perfusion via collaterals. She was discharged with counseling about alcohol cessation and plans for outpatient follow-up. Referral to addiction medicine and psychiatric services was made, but engagement was limited due to non-compliance. The pancreatic head pseudocyst was observed without intervention, as it fluctuated in size on serial imaging but was not independently symptomatic or complicated per current guidelines.

Over the next three months, she had recurrent admissions for alcohol-induced pancreatitis (peak lipase 527 U/L). She continued drinking heavily and developed alcoholic hepatitis (AST 400 U/L, INR 4.0), pulmonary embolism, and reduced left ventricular ejection fraction (45–50%). Serial CT imaging was conducted up to 3 months post-embolization: On 11 July 2024, there were sequelae of prior embolization with interval decrease in pseudocyst size to approximately 3 cm, mild pancreatic edema, and surrounding inflammatory stranding; on 15 July 2024, the pseudocyst measured 3.6 × 2.6 cm with embolization coils causing artifacts; on 15 August 2024, the pseudocyst was thick-walled and well-defined with internal debris, measuring 5.4 × 5.0 cm; on 12 September 2024, the pseudocyst decreased to 1.9 cm with high density, mild proximal pancreatic ductal dilatation, limited splenic vein evaluation due to metallic streak artifact, and no evidence of significant aneurysm recanalization. However, long-term surveillance lapsed due to patient non-compliance. Eight months after the initial embolization, the patient was found unresponsive and in pulseless electrical activity arrest. Return of spontaneous circulation was achieved, but she remained critically hypotensive (blood pressure 61/34 mmHg). Laboratory evaluation revealed severe metabolic acidosis (lactate 19.7 mmol/L), hemoglobin 6.4 g/dL, and lipase 118 U/L. Emergency exploratory laparotomy was initiated within 1–2 h of hospital arrival following resuscitation and revealed massive hemoperitoneum due to rupture of the previously embolized splenic artery pseudoaneurysm (likely via recanalization of the embolized site). The splenic artery was ligated, and a massive transfusion protocol was implemented, which amounted to over 55 units of blood products. Despite aggressive resuscitative measures and surgical intervention, her course was complicated by refractory shock, anuric acute kidney injury, disseminated intravascular coagulation (DIC), and multi-organ failure. After discussions with her family regarding her poor prognosis, care was transitioned to comfort measures, and she subsequently died.

## 3. Discussion

This case highlights a catastrophic failure of long-term management despite a technically successful initial intervention. Several key learning points emerge. Chronic AUD perpetuates pancreatic inflammation that leads to pseudocyst formation and arterial wall erosion. In this patient, relapse directly fueled recurrent pancreatitis flares, which likely promoted vascular instability, recanalization, or new pseudoaneurysm formation, ultimately leading to fatal rupture. This highlights that endovascular therapy alone is insufficient; early, integrated addiction management is essential to mitigate the underlying disease process in high-risk SAP patients [6,7,8]. Multidisciplinary care, including psychiatric support for AUD, is essential to promote abstinence and halt disease progression. The initial gastrointestinal bleed represented a “sentinel bleed,” an event known to precede catastrophic rupture in approximately 40–70% of SAP cases [1,2]. Recognition of a sentinel bleed should trigger not only immediate intervention but also a plan for structured post-embolization surveillance. A reasonable approach, especially in patients with ongoing risk factors like AUD, includes serial CT angiography at intervals such as 1, 3, 6, and 12 months post-procedure [3,4,5,7]. The absence of serial CTA surveillance beyond 3 months, despite recommended protocols, highlights the need for integrated multidisciplinary follow-up to enforce imaging in non-compliant patients. If serial imaging verifies recurrence risks (as partially monitored here with fluctuating pseudocyst size but no early recanalization), elective surgical interventions such as laparotomy with splenectomy and distal pancreatectomy should be considered to prevent rupture. Although covered stent grafts may offer advantages in select cases by maintaining vessel patency and potentially lowering recanalization rates [6,7], coil embolization was selected based on anatomical suitability and institutional expertise, with subsequent imaging confirming initial stability. In cases of rupture or virsungorrhagia, covered stents may provide durable exclusion of the defect and preserve flow, though anatomical suitability varies, as seen in this patient’s tortuous vasculature [6,7]. A notable finding was the marked difference between lipase levels during her pancreatitis flares (449–527 U/L) and the only mildly elevated level at the time of fatal rupture (118 U/L). This suggests that while acute enzymatic activity may initiate pseudoaneurysm formation, the final rupture can occur independently of a severe acute pancreatitis flare. This observation underscores the unreliability of biochemical markers for predicting imminent rupture and reinforces the critical importance of imaging for surveillance and detection of recurrence [7,8]. The patient’s progressive decline from alcohol-related comorbidities, such as alcoholic hepatitis, pulmonary embolism with right heart strain, and cardiac dysfunction; critically limited her ability to tolerate a massive hemorrhagic insult. This case demonstrates that even a technically correct surgical intervention may be un-survivable when physiologic reserve is exhausted by multisystem disease [5,6]. Optimal care requires coordination among gastroenterology, interventional radiology, surgery, and addiction medicine. Vascular intervention alone is insufficient; comprehensive, integrated care is necessary to reduce recurrence risk and improve outcomes [6,7,8,9].

## 4. Conclusions

Fatal splenic artery pseudoaneurysm rupture can occur months after successful coil embolization, without early recanalization, in uncontrolled alcohol-associated chronic pancreatitis. Patient findings: sentinel bleeding warned of instability; AUD relapse fueled recurrent pancreatitis and pseudoaneurysm recurrence; comorbidities reduced physiologic reserve, making intervention futile. Pseudocyst fluctuations and poor surveillance underscore isolated therapy’s limits. Optimal care demands multidisciplinary AUD management, serial CT angiography (1, 3, 6, 12 months), and proactive surgical evaluation.

## Data Availability

The original contributions presented in this study are included in the article. Further inquiries can be directed to the corresponding author.
